# The Geographic Distribution and Natural Variation of the Rice Blast Fungus Avirulence Gene *AVR-Pita1* in Southern China

**DOI:** 10.3390/plants14081210

**Published:** 2025-04-15

**Authors:** Xinwei Chen, Xin Liu, Xiaochun Hu, Zhouyi Tu, Jun Fu, Liping Zhong, Nan Jiang, Yuanzhu Yang

**Affiliations:** 1 Key Laboratory of Southern Rice Innovation & Improvement, Ministry of Agriculture and Rural Affairs, Yuan Longping High-Tech Agriculture Co., Ltd., Changsha 410128, Chinaliuxing9966@126.com (X.L.);; 2 Hunan Engineering Laboratory of Disease and Pest Resistant Rice Breeding, Yuan Longping High-Tech Agriculture Co., Ltd., Changsha 410128, China; 3 College of Life Sciences, Hunan Normal University, Changsha 410081, China; 4 State Key Laboratory of Hybrid Rice, Hunan Hybrid Rice Research Center, Changsha 410125, China; 5 Yuelushan Laboratory, Changsha 410128, China; 6 College of Plant Science & Technology, Huazhong Agricultural University, Wuhan 430070, China

**Keywords:** rice, *Magnaporthe oryzae*, *AVR-Pita1*, *AVR* genes

## Abstract

The avirulence (*AVR*) genes of the filamentous ascomycete fungus *Magnaporthe oryzae* (*M. oryzae*) are known to mutate rapidly under a higher selection pressure, allowing the pathogen to evade recognition by rice *resistance* (*R*) genes. Understanding the geographic distribution and natural variation of *AVR* genes is critical for the rational utilization and prolonging of the effectiveness of *R* genes. In this study, a total of 1060 *M. oryzae* strains collected from 19 rice blast nurseries in 13 provinces across southern China were subjected to presence/absence variation (PAV), genetic variation, and virulence analyses of the *AVR-Pita1* gene. PCR amplification results indicated that *AVR-Pita1* was present in only 57.45% of the blast strains, with significant geographic variation in distribution frequency. Specifically, the highest frequency (100%) was observed in strains from Chengmai, Hainan, while the lowest (1.79%) was observed in strains from Baoshan, Yunnan. A sequencing analysis identified 29 haplotypes of *AVR-Pita1*, characterized by insertions, deletions, and base substitutions. A phylogenetic analysis indicated that haplotypes of *AVR-Pita1* identified in this study were clustered into one clade. A further amino acid sequence analysis of these haplotypes led to the identification of 25 protein variants. Notably, four haplotypes of *AVR-Pita1* exhibited pathogenicity toward its corresponding rice *R* gene, *PtrA*. Additionally, we performed allele profiling of *Ptr* in a collection of elite parental lines that are widely used in rice breeding in southern China and found that the functional *Ptr* alleles (*PtrA*, *PtrB*, and *PtrC*) accounted for over 70%.

## 1. Introduction

Rice blast, caused by the filamentous fungus *Magnaporthe oryzae* (*M. oryzae*), is one of the most damaging diseases affecting rice crops [1,2]. The disease is widely prevalent across rice-growing regions globally, with particularly severe outbreaks in areas such as Asia and Africa posing a significant threat to rice production and food security [2,3]. Developing and planting disease-resistant rice varieties is currently the most economical and effective approach to control this destructive disease [4]. To date, researchers have cloned at least forty *resistance* (*R*) genes in rice, with the majority encoding for nucleotide-binding and leucine-rich repeat domain proteins (NLRs) [5,6,7]. Several of these *R* genes have been extensively deployed in resistance breeding and are crucial in safeguarding rice cultivars against blast disease [8,9]. However, due to the highly variable nature of *M. oryzae*, resistant varieties often experience a decline or complete loss of resistance within three to five years [2,10]. *R* gene-mediated plant resistance follows the gene-for-gene model, wherein R proteins recognize AVR proteins—a subset of effector proteins secreted by phytopathogens to facilitate virulence, to activate defense responses [11]. Genetic alterations in the *AVR* genes can disrupt this specific recognition, a process regarded as a key driver of pathogen virulence evolution that simultaneously compromises the efficacy of corresponding *R* genes [12]. In different ecosystems and during the long-term evolutionary process of natural selection, intense selective pressures have led to continuous coordinated adaptation between phytopathogens and their respective hosts. This adaptation is underpinned by the establishment of an arms race co-evolution relationship between *AVR* genes and *R* genes [13]. To date, 12 *AVR* genes from rice-infecting strains of *M. oryzae* have been cloned, including *AVR-Pita1*, *ACE1*, *AVR-Pia*, *AvrPii*, *AvrPiz-t*, *Avr1-CO39*, *AvrPib*, *AvrPi9*, *AvrPi54*, *AVR-Pias*, *AVR-Mgk1*, and *Avr-Pik* [14,15,16,17,18,19,20,21,22,23,24,25,26]. *Pita*/*AVR-Pita* is the first characterized pair of *R-AVR* in the rice-*M. oryzae* pathosystem [16,27]. *Pita*, located on chromosome 12 of the rice genome, encodes a NLR protein [27]. *AVR*-*Pita* was identified in the telomeric region of chromosome 3 and encodes a putative neutral zinc metalloprotease [16]. Subsequent studies revealed that *AVR*-*Pita* belongs to a gene family comprising at least two additional members, with *AVR*-*Pita* renamed as *AVR-Pita1* [28]. It has long been accepted that *AVR-Pita1* can directly bind to the LRR region of *Pita* [27]. *Ptr/Pita2*, which encodes an atypical R protein containing four Armadillo (ARM) repeats, was subsequently discovered to be tightly linked to *Pita* [29,30]. *Pita2* exhibits resistance to all *Pita*-avirulent strains and some *Pita*-virulent strains [31,32,33]. A recent study indicated that *AVR-Pita* detection relies solely on *Ptr*, rather than *Pita* [34]. The *Pita* resistance is indeed provided by one of the *Ptr* alleles, designated *PtrB*, which recognizes a limited number of *AVR-Pita1* alleles through an indirect mechanism. However, *Pita2* resistance is conferred by *PtrA*, which can detect all tested *AVR-Pita1* alleles. These findings explain why *Pita2* has a broader spectrum of resistance than *Pita*.

The *Ptr* locus has been extensively utilized in breeding blast-resistant cultivars worldwide [35,36,37,38]. However, due to its telomeric location, *AVR-Pita1* frequently exhibits genetic instability [16,39,40,41]. Various genetic mutation and recombination events have been found to be the major driving force to create novel strains virulent toward *Ptr*. For instance, transposon insertion, partial deletion, complete deletion, and sequence variations have been observed in the *AVR-Pita1* sequences among field strains of *M. oryzae* [39,40,42,43,44,45]. Therefore, monitoring the presence and variation of *AVR-Pita1* in the field blast fungal population is essential to predict the effectiveness of *Ptr* alleles.

Southern China, one of the oldest rice cultivation regions in the world, currently serves as the primary rice-producing area in China, accounting for approximately 94% of the national rice planting area and 88% of the total rice yield [46,47]. However, the sub-tropical or tropical humid climates in southern China are extremely conducive for rice blast epidemics. Previously, multiple studies have surveyed *AVR-Pita1* variations in certain rice-growing regions of southern China, but the information provided is fragmented [38,40,45,48]. To fill this information gap, we conducted an investigation into the distribution and genetic variations of the *AVR-Pita1* in 1060 field strains of *M. oryzae* collected from 19 rice blast nurseries situated in 13 provinces across southern China. These nurseries encompass four rice ecological zones. In addition, we assessed the prevalence of *Ptr* alleles in a collection of elite rice parental lines that are frequently used in breeding efforts in southern China. The insights gained from this study are beneficial for conceptualizing breeding and utilization strategies for *Ptr* in the sustainable management of blast diseases in this region.

## 2. Materials and Methods

### 2.1. M. oryzae Strains

**Figure 1:**
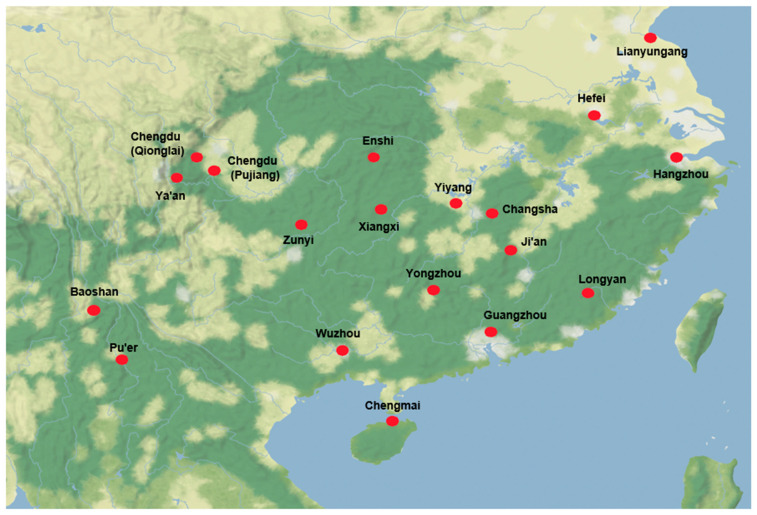
The sampling nurseries of *M. oryzae* strains in southern China. Each red dot represents one sampling nursery. Approximately 50 to 60 single-spore strains from each nursery were isolated.

Naturally infected rice panicles were collected from 19 blast nurseries in 13 provinces across southern China during the period from 2021 to 2023 (Figure 1). Approximately 50 to 60 single-spore strains from each nursery were isolated following the method described by Fei (Table S1) [49]. Strains with clear colonies on rice bran medium (40 g/L rice bran, 20 g/L agar, pH = 6.0–6.5) and typical *M. oryzae* morphological and cultural characteristics were confirmed by PCR using *M. oryzae*-specific primers [50]. All strains were stored at −20 °C on desiccated filter paper.

### 2.2. Plant Materials and Pathogenicity Assay

The *indica* rice cultivar CO39 as a susceptible control and IRBLta2-Pi containing *PtrA* in the background of CO39 were used in this study [51]. Rice cultivars were planted in small pots filled with nutrient-rich soil. Rice seedlings were grown in a greenhouse with a 28/25 °C day/night temperature, and a 16/8 h light/dark photoperiod. At the three-leaf stage, rice seedlings were transferred to the inoculation room, spray-inoculated with *M. oryzae* spore suspensions (1.5 × 10^5^ spores/mL), and kept in darkness (95–100% relative humidity, 22 °C) for 24 h. The inoculated plants were subsequently maintained under a 12/12 h light/dark photoperiod at the same temperature and relative humidity. Disease reaction evaluation was carried out 7 days after inoculation. A scoring system ranging from 0 to 9 based on IRRI’s Standard Evaluation System (SES) was used for the evaluation [52]. The scores of 0 to 3 were classified as resistant, with 4 to 9 as susceptible.

### 2.3. DNA Extraction, PCR Amplification, and Sequencing

Each *M. oryzae* strain was cultured on the rice bran medium at 25 °C for 10 days. Fungal mycelia were harvested and subjected to genomic DNA extraction using the sodium dodecyl sulfate (SDS) method, with the lysis buffer containing 10 mM Tris-HCl (pH 8.0), 1 mM EDTA, 100 mM NaCl, and 2% SDS [53]. Specific primers were used to amplify the *AVR-Pita1* gene (Table S2). A polymerase chain reaction (PCR) was carried out in a 25 μL reaction system: 2.5 μL 10× buffer, 1.0 μL dNTP, 0.5 μL each primer, 1.0 μL DNA template, 0.3 μL Taq enzyme, and 19.2 μL ddH₂O. The thermal cycling protocol included the following: an initial denaturation at 94 °C for 3 min, then 30 cycles of denaturation at 94 °C for 30 s, annealing at 55 °C for 30 s, and extension at 72 °C for 1 min, followed by a final extension at 72 °C for 5 min. PCR products were electrophoresed on a 1% (*w*/*v*) agarose gel stained with ethidium bromide and visualized under UV light. Purified amplicons were subsequently sequenced bidirectionally by Tsingke Biotechnology Co., Ltd. (Beijing, China) using Sanger sequencing.

### 2.4. Phylogenetic Analysis

The DNA sequence of the *AVR-Pita1* gene was assembled and aligned with reference sequences from the National Center for Biotechnology Information (NCBI) database using ClustalW. Subsequently, phylogenetic and molecular evolutionary analyses were conducted using MEGA version 5.0. The phylogenetic tree was specifically constructed using the maximum likelihood method and visualized using MEGA 5.0 software. To assess the robustness of the phylogenetic tree, a bootstrap analysis with 1000 replicates was conducted.

### 2.5. Analysis for the Distribution of Ptr Alleles in Elite Rice Parental Lines

A collection of 1058 elite rice parental lines predominantly utilized in breeding efforts in southern China was subjected to whole-genome sequencing (Table S3). Genomic sequences spanning the *Ptr* (LOC_Os12g18729) coding region were extracted for haplotype analysis according to the *Ptr* classification described by Greenwood et al. [54]. 

## 3. Results

### 3.1. The Presence/Absence Variation of AVR-Pita1 in M. oryzae Strains from Southern China

**Figure 2:**
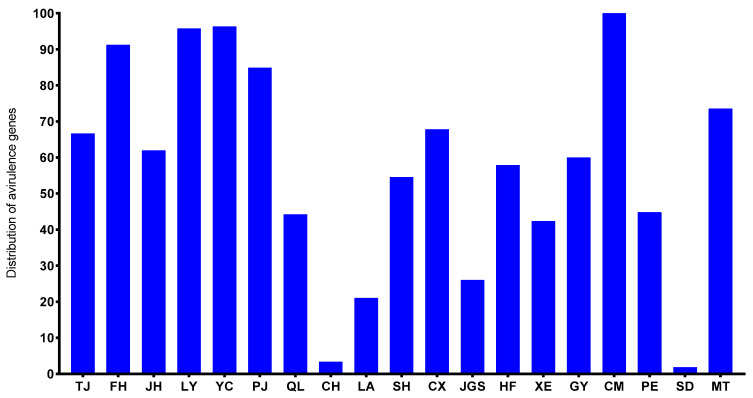
A PAV analysis of *AVR-Pita1* in different nurseries. Approximately 50 to 60 strains from each of the 19 nurseries were analyzed by PCR using *AVR-Pita1*-specific primers. Abbreviations: TJ, Taojiang, Yiyang; FH, Fenghuang, Xiangxi; JH, Jianghua, Yongzhou; LY, Liuyang, Changsha; YC, Yucheng, Ya’an; PJ, Pujiang, Chengdu; QL, Qionglai, Chengdu; CH, Conghua, Guangzhou; LA, Lin’an, Hangzhou; SH, Shanghang, Longyan; CX, Cenxi, Wuzhou; JGS, Jinggangshan, Ji’an; HF, Hefei; XE, Xuanen, Enshi; GY, Ganyu, Lianyungang; CM, Chengmai; PE, Pu’er; SD, Shidian, Baoshan; MT, Meitan, Zunyi.

From 2021 to 2023, a total of 1060 single-spore strains of *M. oryzae* were collected from 19 disease nurseries in southern China (Figure 1, Table 1). These nurseries cover four major rice ecological zones, which exhibit significant differences in environmental conditions and rice cropping patterns. A pair of gene-specific primers was used to amplify *AVR-Pita1* in these field blast strains (Table S2). The presence of a distinct 1076 bp band in electrophoresis analysis served as an indication of the *AVR-Pita1* gene’s existence (Figure S1). PCR amplification analysis demonstrated that the *AVR-Pita1* was detected in 609 strains (57.4% of the total), with its frequency ranging from 1.8% to 100% across different geographical regions (Figure 2). All strains from Chengmai harbored *AVR-Pita1*, followed by Ya’an, Changsha, and Xiangxi, where the detection frequency exceeded 90%. Conversely, *AVR-Pita1* was almost absent in the strains from Baoshan and Guangzhou.

### 3.2. Nucleotide Diversity and the Phylogenetic Analysis of AVR-Pita1

**Figure 3:**
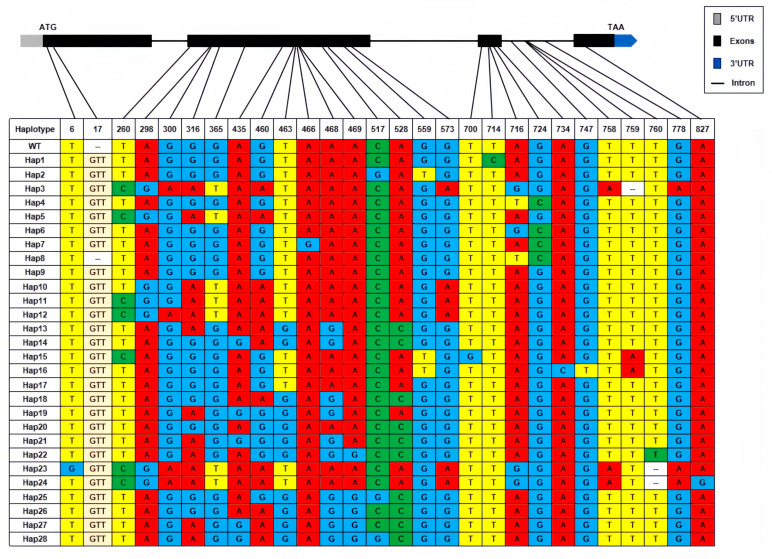
A haplotype analysis of *AVR-Pita1* coding regions from 373 *M. oryzae* strains. The variable sites were marked with different colors.

**Figure 4:**
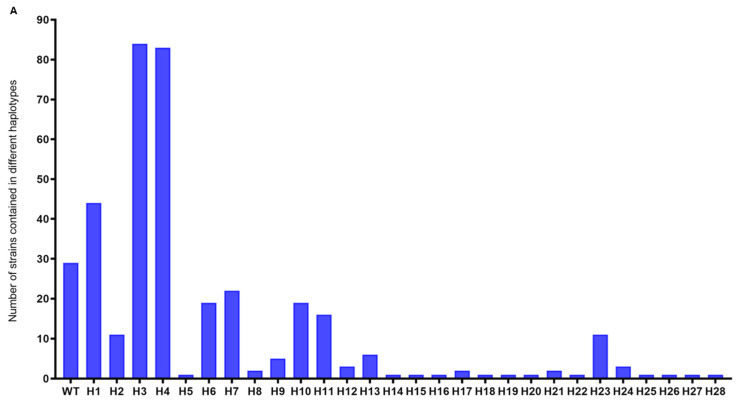

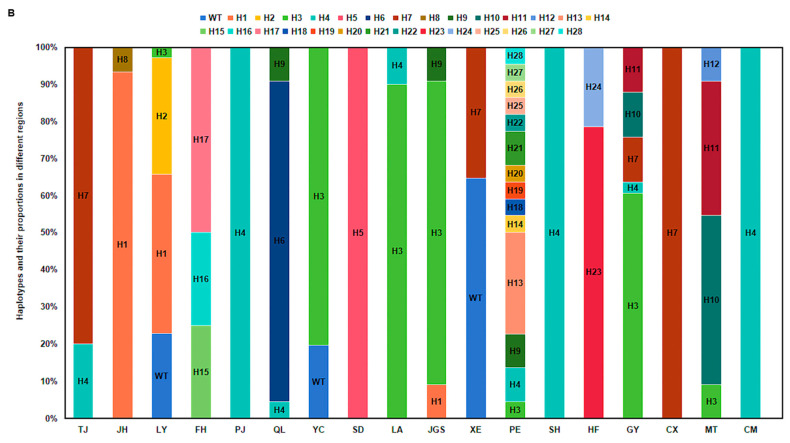
The distribution of different haplotypes of *AVR-Pita1*. (**A**) The number of strains included in different haplotypes. (**B**) Haplotypes and their proportions in different nurseries. WT represents the first characterized haplotypes of *AVR-Pita1* when the gene was cloned. H1–H28 represent 28 haplotypes of *AVR-Pita1* identified in this study. Abbreviations: TJ, Taojiang, Yiyang; JH, Jianghua, Yongzhou; LY, Liuyang, Changsha; FH, Fenghuang, Xiangxi; PJ, Pujiang, Chengdu; QL, Qionglai, Chengdu; YC, Yucheng, Ya’an; SD, Shidian, Baoshan; LA, Lin’an, Hangzhou; JGS, Jinggangshan, Ji’an; XE, Xuanen, Enshi; PE, Pu’er; SH, Shanghang, Longyan; HF, Hefei; GY, Ganyu, Lianyungang; CX, Cenxi, Wuzhou; MT, Meitan, Zunyi; CM, Chengmai.

**Figure 5:**
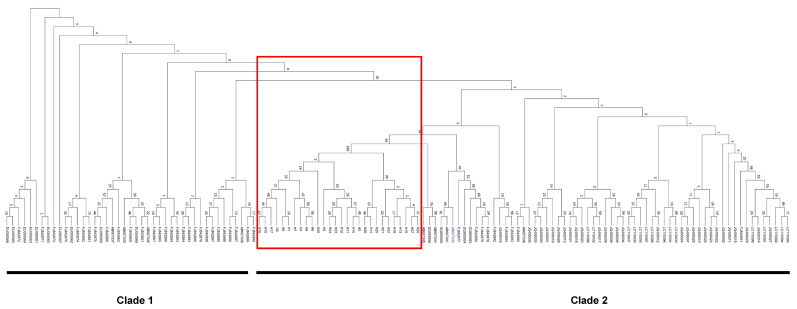
Phylogenetic analysis among *AVR-Pita1* alleles. The *AVR-Pita1* alleles identified in this study are within the red-boxed lines. Accession number shown in blue color represents WT.

To characterize nucleotide variation of *AVR-Pita1*, PCR amplicons from 609 *M. oryzae* strains were sequenced, with 373 successfully yielding high-quality sequences for comparison against the reference sequence (GenBank ID: AF207841). Twenty-eight polymorphic sites were detected, with 23 in exons and 5 in introns (Figure 3). Within the exons, only two SNPs were synonymous. Based on these polymorphisms, the 373 strains were classified into 29 haplotypes, designated H1–H28 and wild-type (WT). H3 (22.52%), H4 (22.25%), and H1 (11.80%) emerged as the dominant haplotypes (Figure 4A). Geographically, H3, H4, and H7 displayed the widest distribution, detected in seven, eight, and four nurseries, respectively, while H5, H8, and H12–H28 were restricted to single nurseries (Figure 4B). Strains from Pu’er exhibited the highest haplotype diversity (14 haplotypes), followed by Lianyungang (5 haplotypes), whereas strains from Chengdu (Pujiang), Baoshan, Longyan, Wuzhou, and Chengmai each harbored only one haplotype (Figure 4B). No successfully sequenced strains were identified from Conghua, Guangzhou. A neighbor-joining phylogenetic tree was constructed with the 29 haplotypes and previously reported *AVR-Pita1* alleles (Table S4). The phylogenetic analysis revealed two major clades: one comprising the haplotypes identified in this study alongside south and southeast Asian strains, and the other containing U.S. strains and strains from Sichuan/Chongqing, China before 2008 (Figure 5). This divergence suggests significant evolutionary shifts in *AVR-Pita1* in southern China over the past decade.

**Figure 6:**
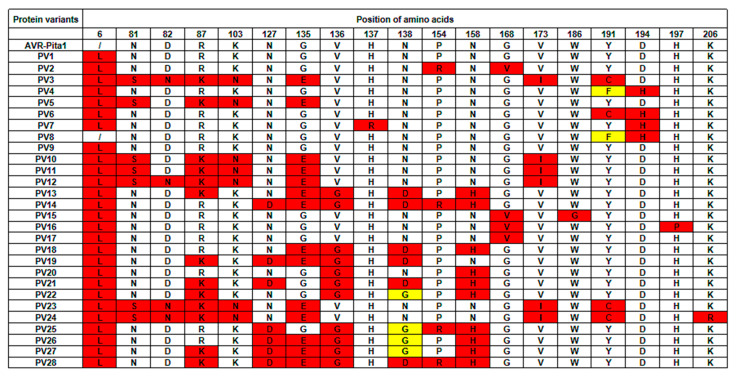
Protein variation among *AVR-Pita1* sequences. Red and yellow represent the translation of protein types different from the reference sequence of *AVR-Pita1*.

A translation of the 29 haplotypes revealed 25 distinct protein variants (Figure 6). Notably, PV3, PV6, PV23, and PV24 exhibited a cysteine-to-tyrosine substitution at position 191—a change critical for evading recognition by *PtrB* [34]. PV4 and PV8 similarly replaced cysteine with phenylalanine at this position. Unlike the previously reported Thai strains, where amino acid variations were predominantly localized to exons 3 and 4, variations in this study clustered primarily in exon 2, accounting for 68.4% of the observed cases [55].

### 3.3. The Virulence Assay of Different AVR-Pita1 Haplotypes

**Figure 7:**
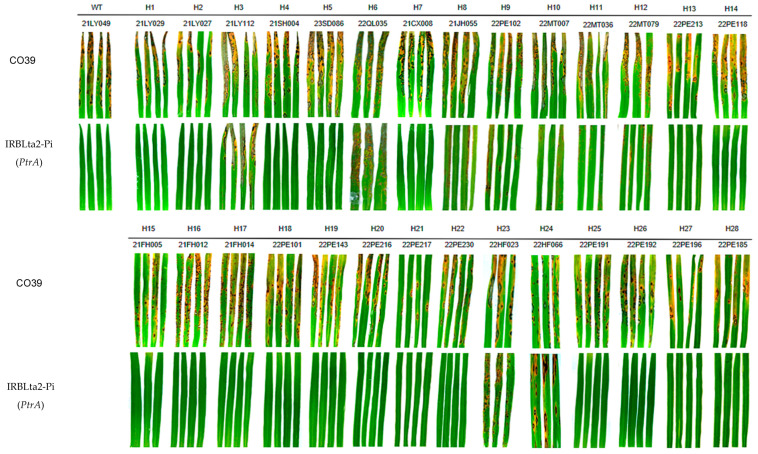
A pathogenicity analysis of different *AVR-Pita1* haplotypes toward *PtrA*. CO39, susceptible control rice cultivar CO39. IRBLta2-Pi, *PtrA* monogenic line. WT represents the first characterized haplotype of *AVR-Pita1* when the gene was cloned. H1–H28 represent 28 haplotypes of *AVR-Pita1* identified in this study. The disease symptoms were photographed 7 days post inoculation.

To evaluate the virulence of distinct *AVR*-*Pita1* haplotypes, we randomly selected one to four representative strains for each haplotype and inoculated them on the monogenic rice blast resistance line IRBLta2-Pi, which carries the *PtrA* allele in the CO39 genetic background (Table S5). Recent studies have found that *PtrA* can recognize all *AVR-Pita1* haplotypes and has a relatively broad-spectrum resistance [34]. In our study, we selected *PtrA* for the inoculation assay. Inoculation assays revealed that four haplotypes, including H3, H6, H23, and H24, successfully infected IRBLta2-Pi, demonstrating virulence (Figure 7; Table S5). All other haplotypes remained avirulent against *PtrA*. The emergence of these virulent *AVR-Pita1* haplotypes highlights challenges for deploying *Ptr*-mediated resistance in rice breeding programs.

### 3.4. The Distribution of the Ptr Alleles in Elite Rice Parental Lines

**Figure 8:**
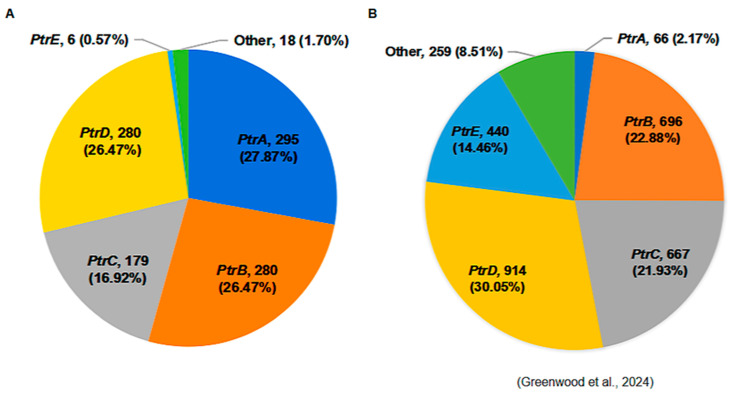
The distribution of *Ptr* alleles in rice cultivars. (**A**) The distribution of the *Ptr* alleles in 1058 elite parental lines used for rice breeding in southern China. (**B**) The distribution of the *Ptr* alleles in 3K rice genomes [54].

To assess the prevalence of *Ptr* alleles in modern Chinese rice cultivars, we analyzed genomic sequences from 1058 elite parental lines that are widely used in rice breeding in southern China. The functional *Ptr* alleles, including *PtrA*, *PtrB*, and *PtrC*, were widely distributed, collectively accounting for 71.3% of the surveyed lines (Figure 8). Notably, *PtrA*, characterized by a relatively broad-spectrum resistance, accounted for 27.9%. This contrasts sharply with its scarcity (2.2%) in the global 3K rice genomes [54]. The elevated frequency of *PtrA* in elite parental lines likely reflects breeders’ conscious or unconscious selection for this allele and implies its historical practical value in cultivation practices within southern rice-growing regions of China.

## 4. Discussion

The rice blast disease remains a perennial threat to Chinese rice production, posing significant risks to food security and profoundly impacting the income and livelihoods of farmers [4]. Southern China, constituting the principal rice-producing zone, is characterized by intricate topography, heterogeneous climatic regimes, diverse rice cultivars, and multifaceted rice cropping systems [47,56,57,58]. These environmental and agronomic variables collectively underpin the evolutionary dynamics of *M. oryzae* populations, driving their high degree of genetic polymorphism and complex spatiotemporal distribution patterns. Monitoring the evolutionary dynamics of *AVR* genes in field populations of *M. oryzae* in southern China is essential for optimizing the sustainability of blast resistance strategies.

In this study, we conducted a large-scale investigation and analysis of the PAV variation and natural variation of the *AVR-Pita1* gene in the field strains of *M. oryzae* from southern China. *AVR-Pita1* was detected in only 57.4% of the tested strains and exhibited extreme spatial variation in its prevalence, ranging from 1.8% in the highland region of Baoshan to 100% in the tropical region of Chengmai. These findings are consistent with prior research [59,60]. For instance, nearly half of the field strains in the Sichuan Basin lacked detectable *AVR-Pita1* [45]. The gene was present in strains from three nurseries but was almost or completely absent in five other rice blast nurseries. In another study, *AVR-Pita1* was detected in only 23 out of 60 Thai strains [60]. In comparison, *AVR-Pi9* and *AVR-Pik* were universally present in these strains. These results suggest that *Ptr*-mediated resistance has been rendered nearly broken down, due to the high loss frequency of *AVR-Pita1* within *M. oryzae* populations. Additionally, *AVR-Pita1* exhibited a high level of genetic variation. We identified 28 polymorphic sites within the *AVR-Pita1* locus in the strains from southern China, which generated 29 distinct haplotypes. Similarly, a high haplotype diversity in *AVR-Pita1* was also reported in strains from Thailand, Vietnam, the USA, and Colombia [43,55,61]. The telomeric location of *AVR-Pita1* on chromosomes is likely a primary contributor to such genetic instability [16,28]. Meanwhile, the evolutionary dynamics of the *AVR-Pita1* gene in *M. oryzae* populations are strongly influenced by host–pathogen co-evolution, with multiple studies demonstrating that positive selection pressure drives its genetic diversity [39,43,55].

Based on the phylogenetic analysis, we found the 29 *AVR-Pita1* haplotypes identified in this study form a clade along with strains from south and southeast Asian countries, such as Thailand, the Philippines, Indonesia, and India, while historical strains from the United States, Sichuan, and Chongqing in China before 2008 cluster into another clade. This result is in agreement with previous findings that emphasized the significant role of geographical location in the distribution of genetic variations in *AVR* genes [28,43]. This differentiation suggests significant spatiotemporal heterogeneity in the evolution of *AVR-Pita1*, which may result from the different environments and rice genotypes. Southeast Asia, including the Yunnan Province, is considered both the center of origin and genetic diversity of the rice-infecting lineage of *M. oryzae* [62,63,64,65,66,67]. Globally, the rice-infecting lineage is subdivided into four distinct lineages. Notably, lineage 1, which is predominantly distributed in East and Southeast Asia, is unique in exhibiting signatures of recombination and sexual reproduction [67]. Within China, Yunnan Province serves as a primary region for the distribution of lineage 1, which may account for the high haplotype diversity observed in strains from Pu’er. In addition, this could also correlate with the extensive presence of wild rice and genetically diverse landraces in the region, potentially leading to a reduced selection pressure on the pathogen. The elevated frequency of functional *Ptr* alleles (*PtrA*, *PtrB*, and *PtrC*) in elite rice parental lines frequently used in southern China (71.3%) reflects both conscious breeding efforts and unconscious selection pressures. The disproportionate representation of *PtrA* (27.9%) compared to global germplasm (2.2% in 3K rice genomes) highlights its historical utility in southern China. It is particularly noteworthy that in our breeding practices, *PtrA* has been strategically deployed as a key target gene for blast resistance, either as a standalone component or pyramided with other *R* genes (e.g., *Pigm* and *Pi2*). However, the widespread deployment of functional *Ptr* alleles (particularly *PtrA*) has imposed intense selection pressure on *AVR-Pita1*, driving its accelerated genetic diversification through mutations. Ultimately, this leads to the loss of *AVR-Pita1*’s avirulence function and the erosion of *Ptr* resistance.

In conclusion, our findings on the geographic distribution, genetic diversity, and virulence profiles of *AVR-Pita1* underscore the complexity of pathogen adaptation in southern China. This study has certain limitations: (1) the sampling window (2021–2023) may not capture emerging variations; (2) a potential sampling bias in regional pathogen reservoirs. Future efforts must prioritize real-time spatiotemporal dynamic pathogen surveillance, diversified breeding strategies, and adaptive management frameworks in rice breeding programs [68,69].

## Figures and Tables

**Table 1:** Detailed information on rice blast fungus sample collection sites.

Province	City/Prefecture	County/District	Abbreviation	Ecological Zone
Hunan	Changsha	Liuyang	LY	The middle and lower reaches of the Yangtze River
	Yiyang	Taojiang	TJ	The middle and lower reaches of the Yangtze River
	Xiangxi	Fenghuang	FH	Wuling Mountainous Area
	Yongzhou	Jianghua	JH	The middle and lower reaches of the Yangtze River
Sichuan	Chengdu	Pujiang	PJ	The upper reaches of the Yangtze River
		Qionglai	QL	The upper reaches of the Yangtze River
	Ya’an	Yucheng	YC	The upper reaches of the Yangtze River
Jiangsu	Lianyungang	Ganyu	GY	The middle and lower reaches of the Yangtze River
Zhejiang	Hangzhou	Lin’an	LA	The middle and lower reaches of the Yangtze River
Anhui	Hefei	/	HF	The middle and lower reaches of the Yangtze River
Fujian	Longyan	Shanghang	SH	South China
Guangdong	Guangzhou	Conghua	CH	South China
Guangxi	Wuzhou	Cenxi	CX	South China
Jiangxi	Ji’an	Jinggangshan	JGS	The middle and lower reaches of the Yangtze River
Hubei	Enshi	Xuan’en	XE	Wuling Mountainous Area
Hainan	/	Chengmai	CM	South China
Yunnan	Pu’er	/	PE	The upper reaches of the Yangtze River
	Baoshan	ShiDian	SD	The upper reaches of the Yangtze River
Guizhou	Zunyi	Meitan	MT	The upper reaches of the Yangtze River

## Data Availability

The original contributions presented in this study are included in the article/Supplementary Material. Further inquiries can be directed to the corresponding authors.

## Supplementary Materials

The following supporting information can be downloaded at https://www.mdpi.com/article/10.3390/plants14081210/s1; Figure S1. PCR amplification of *AVR-Pita1*. (A) The position of the primer pair AVR-Pita1F/AVR-Pita1R used to detect the presence of the *AVR-Pita1* locus. (B) Amplification results of some regions in the CDS region. The electrophoresis pattern shows the amplification results of some strains from Liuyang (LY) area. For those with the *AVR-Pita1* gene locus, a bright band sized 1076bp can be amplified. Table S1. Rice blast strains used in this study. Table S2. The information of primers used in this study. Table S3. Haplotype analysis of Chinese backbone rice parents for *Ptr*. Table S4. Information of strains and sequence IDs for phylogenetic analysis. Table S5. The virulence of different *AVR-Pita1* haplotypes to *PtrA*.
