# Supplementary material for: The Geographic Distribution and Natural Variation of the Rice Blast Fungus Avirulence Gene AVR-Pita1 in Southern China
Source: Plants (Basel). 2025 Apr 15;14(8):1210. doi: 10.3390/plants14081210 (PMC12030372; doi:10.3390/plants14081210)

Supplemental Figure S1. PCR amplification of *AVR-Pita1*. (A) The position of the primer pair AVR-Pita1F/AVR-Pita1R used to detect the presence of the *AVR-Pita1* locus. **(B)** Amplification results of some regions in the CDS region. The electrophoresis pattern shows the amplification results of some strains from Liuyang (LY) area. For those with the *AVR-Pita1* gene locus, a bright band sized 1076bp can be amplified.

A

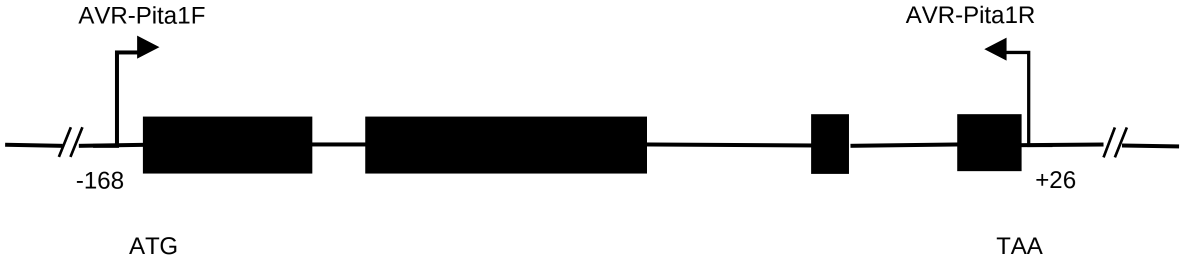

B

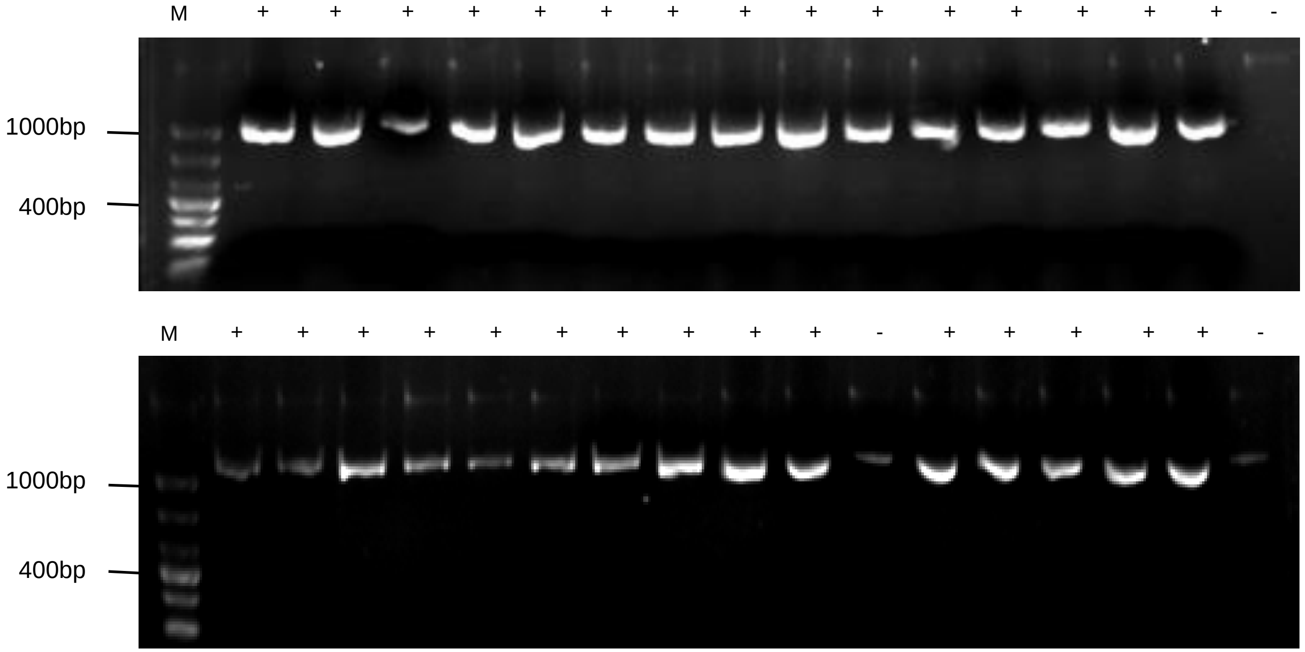

Supplement: Supplementary file 1 [file plants-14-01210-s001.zip › Figure S1.pdf]
